# Role of long non-coding RNA TP73-AS1 in cancer

**DOI:** 10.1042/BSR20192274

**Published:** 2019-10-18

**Authors:** Caizhi Chen, Long Shu, Wen Zou

**Affiliations:** Department of Oncology, The Second Xiangya Hospital of Central South University, Changsha 410000, Hunan, China

**Keywords:** cancer, long non-coding RNA TP73-AS1, tumorigenesis

## Abstract

Cancer incidence rate has increased so much that it is the second leading cause of deaths worldwide after cardiovascular diseases. Sensitive and specific biomarkers are needed for an early diagnosis of cancer and in-time treatment. Recent studies have found that long non-coding RNAs (lncRNAs) participate in cancer tumorigenesis. LncRNA P73 antisense RNA 1T (TP73-AS1), also known as KIAA0495 and p53-dependent apoptosis modulator (PDAM), is located in human chromosomal band 1p36.32 and plays a crucial role in many different carcinomas. This review summarizes current findings on the role of TP73-AS1 and its signaling pathways in various cancers, including glioma, esophageal squamous cell carcinoma (ESCC), hepatocellular carcinoma (HCC), colorectal cancer (CRC), osteosarcoma, gastric cancer (GC), clear cell renal cell carcinoma (ccRCC), breast cancer (BC), bladder cancer, ovarian cancer, cholangiocarcinoma (CCA), lung cancer, and pancreatic cancer. Its aberrant expression generally correlates with clinicopathological characterization of patients. Moreover, TP73-AS1 regulates proliferation, migration, invasion, apoptosis, and chemoresistance cancer mechanisms, both *in vivo* and *in vitro*, through different signaling pathways. Therefore, TP73-AS1 may be considered as a marker for diagnosis and prognosis, also as a target for cancer treatment.

## Introduction

Cancer incidence rate has increased so much that it is the second leading cause of deaths around the world after angiocardiopathy [1]. In 2015, it was estimated that there would be approximately 4292000 new cancer cases and 2814000 cancer deaths in China [2]. Whereas, approximately 18.1 million new cancer cases and 9.6 million cancer deaths due to 36 different cancers occurred in 185 countries around the world in 2018 [3]. Moreover, 1762450 new cancer cases and 606880 cancer deaths are expected in 2019 in the United States [4]. With the increasing burden of cancer, sensitive biomarkers are needed to detect and diagnose cancer at an early stage, to reduce both morbidity and mortality during treatment. Long non-coding RNA (lncRNAs) are among the most sensitive and specific cancer biomarkers known.

LncRNAs are transcripts longer than 200 nucleotides transcribed by RNA polymerase II (RNA Pol II), but not translated into proteins [5]. In the past, they were considered as transcriptional noise; only 2% of the human genome is formed by protein-coding genes [6]. However, in recent years, an increasing number of studies have found that lncRNAs are involved in the regulation of the development of various diseases, especially cancer. The results indicated that lncRNAs implicated in epigenetic regulation, genomic instability, tumor progression, drug resistance, and gene therapy in cancer. Moreover, lncRNAs could be used as the diagnostic and prognostic biomarkers in human malignancies [7].

LncRNAs may work in different ways within the cell: (1) as scaffolds to collect and activate protein complexes [8]; (2) as enhancers for the recruitment of protein complexes, inducing chromosomal cyclization and the transcription of target genes [9]; (3) as decoys, capturing inhibitory complexes, thus preventing inhibition of gene expression [10]; (4) as guides for transcriptional repressors toward targeting genes, leading to transcriptional repression [11]; (5) as a competitive endogenous RNA (ceRNA) that functions as a microRNA (miRNA) sponge to avoid the degeneration of mRNA, thereby increasing the expression of specific transcripts [12]; (6) as transcription inhibitors through lncRNA–DNA binding, thus inducing the stalling of RNA Pol II [13].

**Figure 1:**
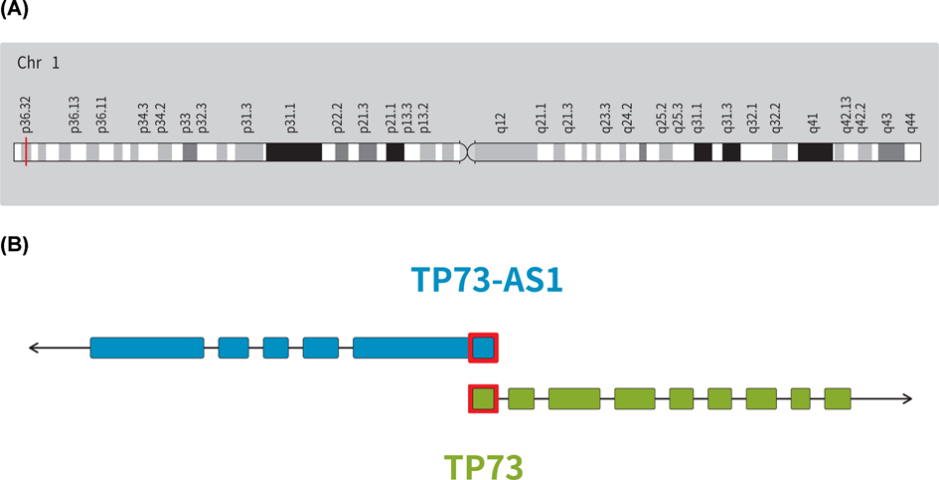
The location of TP73-AS1 on the chromosomal band and its relationship with TP73 (**A**) TP73-AS1 location and (**B**) schematic diagram of the sequence relationship between TP73-AS1 and *TP73* gene (black arrow for transcription direction, red border for overlapping region).

LncRNA P73 antisense RNA 1T (TP73-AS1), also known as KIAA0495 or p53-dependent apoptosis modulator (PDAM), is located on the human chromosomal band 1p36.32 and has an approximately 216-bp overlap with the untranslated region of an adjacent gene, *TP73* (p73), that is transcribed from the opposite strand starting from its own promoter (Figure 1A). Genomic structure analysis has showed that TP73-AS1 contains six exons with a length of 4690 bp. TP73 is a member of the tumor protein 53 (TP53) family of transcription factors, it contains nine exons and 636 amino acids (Figure 1B) [14]. TP73-AS1 covers substantial portions of TP73, suggesting that TP73-AS1 may function by post-transcriptional regulation of TP73 gene expression [15]. Many years of research has shown that TP73-AS1 plays a key role in the development of cancer. For this review, we searched Pubmed and Web of Science databases using ‘TP73-AS1’, ‘KIAA0495’, and ‘PDAM’ as key words and then selected those articles associated with cancer. Based on selected articles, we summarize the role of TP73-AS1 in different cancers, including dysregulated expression, biological functions, related molecules, and associated clinical characteristics (Table 1).

**Table 1:** The role of TP73-AS1 in human cancers/tumors

Cancer/tumor	Source of samples	Aberrant expression	Associated clinical features	Biological functions	Target molecules of TP73 AS1	REF
Glioma	Human patient/tumor samples and *in vitro* assays	Down-regulated	1p/19q co-deletion, tumor location in frontal lobe	Promote chemoresistance to cisplatin	BCL2, BCL2L1	[16]
		Up-regulated		Promote cell proliferation and invasion	miR-124/p53 (iASPP)	[17]
		Up-regulated	WHO stage, OS and tumor size	Promote cell proliferation and invasion	miR-142/HMGB1/RAGE	[18]
ESCC	Human patient/tumor samples and *in vitro* assays	Up-regulated	TNM stage and tumor location	Promote proliferation and chemoresistance to 5-FU, cisplatin, inhibit apoptosis	BDH2	[19]
Hepatocellular carcinoma	Human patient/tumor samples and *in vitro* assays	Up-regulated	TNM stage, tumor size, tumor nodule number and poor prognosis	Promote cell proliferation	miR-200a/HMGB1/RAGE	[20]
Colorectal cancer	Human patient/tumor samples and *in vitro* assays	Up-regulated	Metastasis and clinical stage	Promote cell growth, proliferation, migration, and invasion	miR-194/TGF-α	[21]
		Down-regulated	Clinical stage, OS and DFS	Promote cell proliferation, inhibit apoptosis	miR-103/PTEM	[22]
Osteosarcoma	Human patient/tumor samples and *in vitro* assays	Up-regulated	Clinical stage, tumor size, metastasis, OS, histological grade	Promote cell proliferation, migration and invasion		[23]
					miR-142/Rac1	[24]
Gastric cancer	Human patient/tumor samples and *in vitro* assays	Up-regulated	TNM stage, LNM, DM, OS, depth of invasion, and differentiation	Promoted cell proliferation, invasion, metastasis and inhibited apoptosis, decreased chemosensitivity to cisplatin	miR-194-5p/SDAD1	[25]
					HMGB1/RAGE	[26]
					EMT/Bcl-2/caspase-3	[27]
Clear cell renal cell carcinoma	Human patient/tumor samples and *in vitro* assays	Up-regulated	Tumor metastasis, tumor size, TNM stage and poor prognosis	Promote cell proliferation, invasion, inhibit apoptosis	KISS/EZH2, PI3K/Akt/mTOR	[28]
Breast cancer	Human patient/tumor samples and *in vitro* assays	Up-regulated	Tumor size, LNM, TNM stage, and OS	Promote vasculogenic mimicry, cell proliferation, invasion and migration	miR-490-3p/TWIST1	[29]
					miR-200a/TFAM	[30]
					miR-200a/ZEB1	[31]
Bladder cancer	Human patient/tumor samples and *in vitro* assays	Down-regulated	Tumor stage,TNM stage, OS and DFS	Promote cell growth, cell cycle, migration and invasion, inhibit apoptosis	EMT	[32]
Ovarian cancer	Human patient/tumor samples and *in vitro* assays	Up-regulated	Tumor size, FIGO stage and LNM	Promote cell proliferation, invasion and migration	MMP2, MMP9	[33]
Cholangiocarcinoma	Human patient/tumor samples and *in vitro* assays	Up-regulated	Tumor size and TNM stage	Promote cell proliferation, migration and invasion, inhibit apoptosis		[34]
Non-small cell lung cancer	Human patient/tumor samples and *in vitro* assays	Up-regulated	Tumor size, TNM stage, LNM and poor prognosis	Promote cell proliferation, tumor growth and cycle progression	miR-449a/EZH2	[35]
Lung adenocarcinoma	human patient/tumor samples and *in vitro* assays	Up-regulated	TNM stage, tumor size, LNM and OS	Promote cell proliferation, migration, and invasion, repress apoptosis	PI3K/AKT	[36]
Pancreatic cancer	Human patient/tumor samples and *in vitro* assays	Up-regulated	TNM stage, LNM, OS	Promote migration and invasion	miR-141-3p/BDH2	[37]

Abbreviations: BCL2L1, BCL2-like 1; BDH2, butyrate dehydrogenase 2; DFS, disease-free survival; DM, distant metastasis; EMT, epithelial-to-mesenchymal transition; ESCC, esophageal squamous cell carcinoma; EZH2, enhancer of zeste homolog 2; FIGO, Federation of Gynecology and Obstetrics; HMGB1, high mobility group box 1 protein; iASPP, inhibitor of apoptosis stimulating protein of p53; LNM, lymph node metastasis; MMP, matrix metallopeptidase; OS, overall survival; Rac1, Ras-related C3 toxin substrate 1; RAGE, receptor for advanced glycation end product; REF, reference; TFAM, mitochondrial transcription factor A; TNM, tumor lymph node metastasis; ZEB1, zinc finger E-box binding homeobox 1.

### TP73-AS1 in cancer

#### Glioma

TP73-AS1 was found to be down-regulated in oligodendroglioma; its low levels were related to an 1p/19q co-deletion and to tumor location. This implies that both the loss of chromosome 1p and epigenetic modifications were the main mechanisms leading to TP73-AS1 down-regulation. Moreover, it was confirmed that a knockdown of PDAM in glioma cells induced cisplatin resistance mainly because of the up-regulation of the anti-apoptotic gene, BCL2-like 1 (*BCL2L1*) [16]. In contrast, a study by Xiao et al. [17] showed that the expression of TP73-AS1 was up-regulated in glioma tissue samples and that knocking down TP73-AS1 could suppress glioma cell proliferation and invasion. Mechanistically, TP73-AS1 might be acting as a ceRNA to regulate inhibitor of apoptosis stimulating protein of p53 (iASPP) expression through sponging miR-124. Furthermore, Zhang et al. [18] found that TP73-AS1 was significantly up-regulated in brain glioma clinical tissue specimens and cells, which was linked to tumor size, WHO stage, overall survival (OS), and poor prognosis. Silencing of TP73-AS1 inhibited glioma cell proliferation, invasion and high mobility group box 1 protein (HMGB1) protein expression. Further investigation revealed that TP73-AS1 competed with HMGB1 for miR-142 binding to modulate HMGB1 expression via an miR-142 sponge, which participated in the modulation of glioma cell proliferation and invasion. In summary, TP73-AS1 may be regarded as a novel therapeutic biomarker for glioma treatment, but further studies are needed to confirm this conclusion.

### Esophageal squamous cell carcinoma

TP73-AS1 and butyrate dehydrogenase 2 (BDH2) were both up-regulated in esophageal squamous cell carcinoma (ESCC) tissue samples, while the expression of TP73-AS1 was obviously correlated both with tumor lymph node metastasis (TNM) stage and tumor location, BDH2 expression was only statistically associated with TNM stage. Silencing of TP73-AS1 induced ESCC apoptosis and inhibited proliferation, whereas overexpression of BDH2 could reverse this process via the caspase-3 pathway. Moreover, it was confirmed that silencing of TP73-AS1 reduced the average tumor size in mice. In addition, knocking down TP73-AS1 or BDH2 increased the chemosensitivity of ESCC to cisplatin and 5-FU [19]. These results indicate that lncRNA TP73-AS1 could be used as a novel target for ESCC treatments.

### Hepatocellular carcinoma

TP73-AS1 was dramatically up-regulated in hepatocellular carcinoma (HCC) cell lines and tissues; up-regulation correlated with TNM stage, tumor size, tumor nodule number, OS, and poor prognosis. Additionally, silencing of TP73-AS1 inhibited HCC cell proliferation. It has also been demonstrated that TP73-AS1 suppresses miR-200a to facilitate HCC cell proliferation via the HMGB1/receptor for advanced glycation end product (RAGE) signaling pathway [20]. Thus, TP73-AS1 may be playing an important role as a modulator of tumor growth in HCC and could be regarded as a potential biomarker for HCC treatment.

### Colorectal cancer

As expected, TP73-AS1 was found to be up-regulated in colorectal cancer (CRC) cell lines as well as in CRC tissue samples; its overexpression was linked to advanced clinical stages and metastasis. Furthermore, knocking down TP73-AS1 markedly depresses CRC cell proliferation, migration, and invasion *in vitro* as well as tumor growth *in vivo*. Mechanistically, TP73-AS1 modulated CRC tumorigenesis by regulating the expression of transforming growth factor α (TGFα), acting as a ceRNA to sponge miR-194 [21]. However, other studies showed that TP73-AS1 was down- regulated in CRC tissue samples and cell lines; here, low expression levels were correlated to TNM stage, prognosis, OS, and disease-free survival (DFS) of patients with CRC. Additionally, TP73-AS1 overexpression significantly promoted CRC apoptosis and inhibited cell growth. Functionally, TP73-AS1 facilitated CRC cell proliferation by inducing phosphate and tension homology deleted on chromsome ten (PTEN) expression by binding to miR-103 [22]. All these results have provided a foresight into the potential role of a TP73-AS1 signaling pathway mediating CRC.

### Osteosarcoma

TP73-AS1 was found to be up-regulated in osteosarcoma cell lines and tissue samples; its overexpression was significantly linked to clinical stage, tumor size, metastasis, histological grade, OS, and poor prognosis. Moreover, a knockdown mutation of TP73-AS1 suppressed cell survival, migration, and invasion, inducing cell cycle arrest in osteosarcoma *in vitro* [23]. Mechanistically, TP73-AS1 silencing depressed osteosarcoma tumor growth *in vivo* as well as cell proliferation and invasion *in vitro* via regulating the miR-142/Ras-related C3 toxin substrate 1 (Rac1) pathway [24]. In conclusion, TP73-AS1 might be regarded as a carcinogenic lncRNA involved in the development of osteosarcoma, offering a novel therapeutic insight for osteosarcoma treatment.

### Gastric cancer

Several studies have shown that TP73-AS1 is up-regulated in gastric cancer (GC) cell lines as well as in tissue samples; expression clearly correlated with TNM stages, lymph node metastasis, distant metastasis, depth of invasion, differentiation, and OS. Moreover, TP73-AS1 facilitated cell proliferation, invasion, and metastasis, while inhibited apoptosis and provoked a decreased chemosensitivity of GC cells to cisplatin. Mechanistically, TP73-AS1 modulated the tumor progression of GC cells through several signaling pathways, involving targeting miR-194-5p/SDAD1, down-regulation of HMGB1/RAGE, and regulating Bcl-2/caspase-3 or reversing epithelial-to-mesenchymal transition (EMT) [25–27]. Therefore, TP73-AS1 might function as a GC oncogenic factor and provide an effective prognostic/therapeutic target for patients with GC.

### Clear cell renal cell carcinoma

TP73-AS1 was up-regulated in clear cell renal cell carcinoma (ccRCC) tumor cells compared with adjacent normal tissues. High expression levels were associated with TNM stage, tumor size, metastasis, and poor prognosis. Moreover, a TP73-AS1 knockdown suppressed cell proliferation and invasion, and facilitated apoptosis, whereas overexpression of TP73-AS1 reversed this progression. Furthermore, It has been shown that TP73-AS1 regulates cell proliferation and apoptosis via the down-regulation of KISS1 expression and the PI3K/Akt/mTOR pathway, respectively [28]. Thus, TP73-AS1 plays a crucial role in the tumorigenesis of ccRCC and may offer a novel therapeutic biomarker for this disease.

### Breast cancer

Both in breast cancer (BC) tissue samples and cell lines, TP73-AS1 has been found to be up-regulated and this overexpression has been related to several BC clinicopathologic characterizations, such as TNM stage, tumor size, lymph node metastasis, and oOS In contrast, TP73-AS1 silencing suppressed vasculogenic mimicry, invasion, migration, and inhibited BC cell proliferation. Mechanistically, it was shown that several pathways participate in those biological functions altered after the knockdown of TP73-AS1. Targeting the miR490-3p/TWIST1 axis, regulating the expression of mitochondrial transcription factor A (TFAM) by depressing miR-200a, and forming the TP73-AS1/miR-200a/zinc finger E-box binding homeobox 1 (ZEB1) modulating loop in BC cells [29–31], are among these mechanisms. All these findings suggest that TP73-AS1 might provide an insight into BC management.

### Bladder cancer

Studies have shown that TP73-AS1 is down-regulated in bladder cancer tissues and cell lines, which is significantly linked to tumor stage, TNM stage, OS, and progression-free survival (PFS). Mechanistically, the overexpression of TP73-AS1 could stimulate cell apoptosis and depress tumor growth, arrest cell cycle, and weaken cell invasion and migration *in vitro* by the suppression of EMT [32]. These results indicate that lncRNA TP73-AS1 might serve as a tumor suppressor since a low expression level contributes to the progression of bladder cancer, offering a prospective therapeutic target for the treatment of this disease.

### Ovarian cancer

Expression studies have shown that TP73-AS1 is markedly up-regulated in tissue samples and cell lines of ovarian cancer. High expression levels have also been correlated with FIGO stage, tumor size, and lymph node metastasis. Silencing of TP73-AS1 inhibited cell proliferation, migration, and invasion, while overexpression stimulated progression *in vitro*. Furthermore, the knockdown of the TP73-AS1 gene depressed tumor growth in mice. Mechanistically, down-regulation of matrix metallopeptidase (MMP) 2 (*MMP2*) and 9 (*MMP9*) genes reduced the influence of TP73-AS1 overexpression in cell migration and invasion, but further investigation is still needed to unveil the mechanism of action of these genes in ovarian cancer progression [33]. Nevertheless, these results acknowledge the carcinogenic role of TP73-AS1 in ovarian cancer; TP73-AS1 might be a target in the search for new ways to fight against this disease.

### Cholangiocarcinoma

When studying cholangiocarcinoma (CCA) tissue samples and cell lines, it was found that TP73-AS1 was up-regulated; this overexpression was markedly correlated both with TNM stage and tumor size. Meanwhile, a knockdown mutation of the TP73-AS1 gene inhibited tumor growth both *in vitro* and *in vivo*. In addition, knocking down TP73-AS1 promoted apoptosis through the activation of the caspase-3 and caspase-9 pathways. Furthermore, TP73-AS1 could potentiality stimulate invasion and migration capacities of CCA cells [34]. In summary, the study concluded that TP73-AS1 might be a novel therapeutic biomarker for CCA treatment.

### Lung cancer

TP73-AS1 was up-regulated in both cell lines and tissue samples of non-small cell lung cancer (NSCLC) and lung adenocarcinoma (LAD). Overexpression was linked to TNM stage, tumor size, lymph node metastasis, and poor prognosis. Additionally, a knockdown mutation of TP73-AS1 inhibited NSCLC cell proliferation *in vitro* and inhibited tumor growth and cycle development *in vivo* as well as *in vitro*. In LAD, TP73-AS1 facilitates cell proliferation, promotes cell migration and invasion, but represses apoptosis *in vitro*. Moreover, silencing TP73-AS1 depressed LAD tumor growth and metastasis *in vivo*. Mechanistically, the molecular pathway TP73-AS1/miR-449a/enhancer of zeste homolog 2 (EZH2) promotes NSCLC tumorigenesis via epigenetic modulation, whereas TP73-AS1 promotes the progression of LAD through the activation of the PI3K/AKT signaling pathway [35,36]. Collectively, TP73-AS1 might be a promising therapeutic and prognostic indicator both for LAD and NSCLC.

### Pancreatic cancer

Studies have demonstrated that TP73-AS1 is up-regulated in pancreatic cancer tissue and cells and that high expression levels are markedly related to the TNM stage and lymph node metastasis, as well as to OS. Silencing of TP73-AS1 suppresses pancreatic cancer migration and invasion. Furthermore, TP73-AS1 actively regulates BDH2 by modulating miR-141, which participates in pancreatic cancer progression [37]. In conclusion, TP73-AS1 could be regarded as a predictor and a novel target for the therapy and prognosis of pancreatic cancer.

## Conclusions and future perspectives

**Figure 2:**
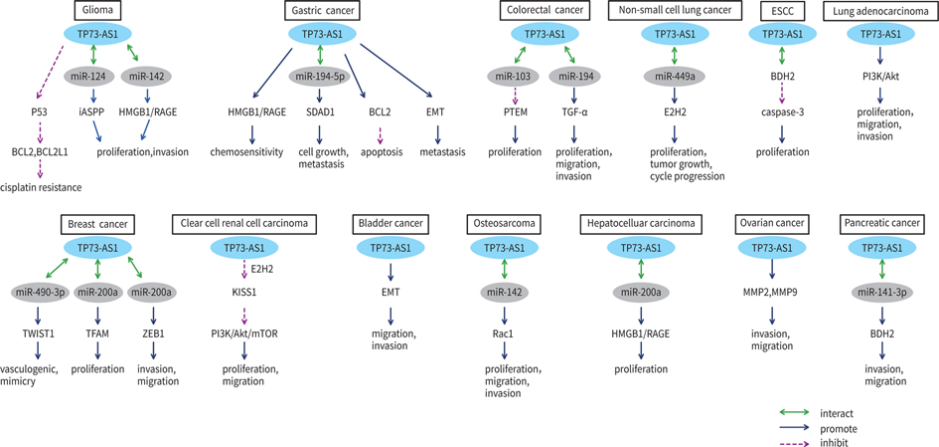
The molecular mechanisms of TP73-AS1 in various human cancers .

**Figure 3:**
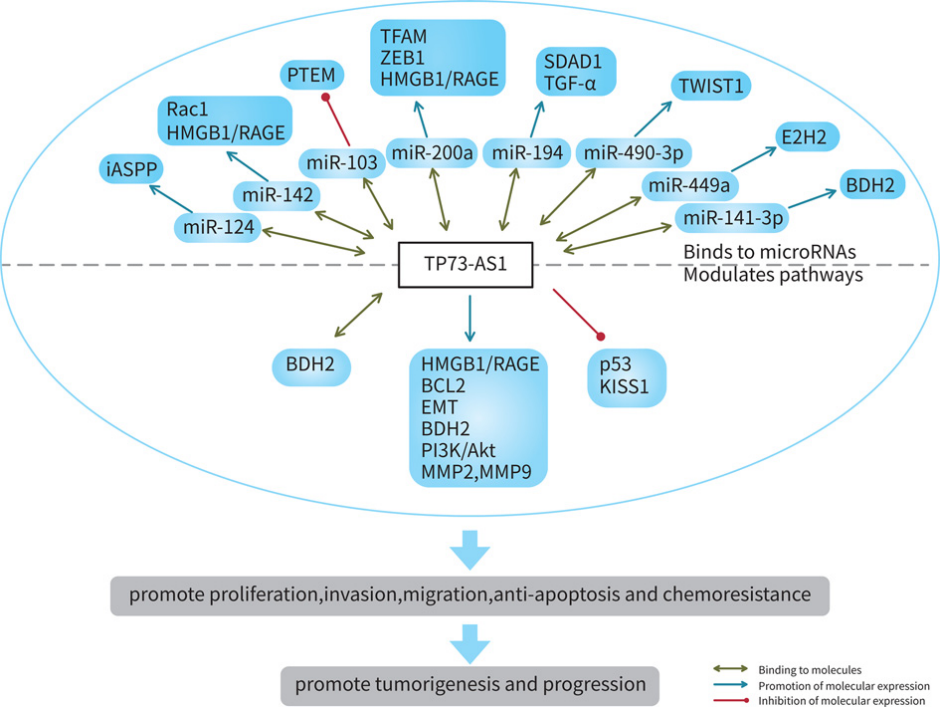
Main mechanisms of lncRNA TP73-AS1 in tumorigenesis and progression .

LncRNAs play a crucial role in the progression of tumors. The recently discovered lncRNA TP73-AS1 is highly expressed in most tumor tissues and cell lines studied, except bladder cancer. Its abnormal expression is statistically correlated with clinicopathological characteristics of cancer, such as TNM stage, tumor size, lymph node metastasis, and prognosis. As a tumor promoter, lncRNA TP73-AS1 also participates in the regulation of a variety of cellular biological behaviors; it promotes cell proliferation, invasion, metastasis, chemotherapy sensitivity, and inhibition of apoptosis. In terms of the mechanisms involved in all these processes, it is known that TP73-AS1 often competes with miRNAs to regulate downstream signaling molecules, redirecting the cell metabolism to a completely different signaling pathway or directly acting on downstream targets leading to an abnormal expression of the *TP73-AS1* gene, activating a series of biological functions (Figures 2 and 3). Consequently, TP73-AS1 may be considered as a marker of both cancer diagnosis and prognosis, and as a target for the treatment of several cancers.

The function and molecular signaling pathways involving TP73-AS1 in various cancers have been studied thoroughly. In addition, methylated TP73-AS1 is frequently detected in cancer cell lines of multiple myeloma, but this methylation does not have a significant correlation with tumor pathogenesis and progression [38]. In retinoblastoma (Rb) tumor tissues, it was seen that TP73-AS1 was up-regulated while miR-139-3p was down-regulated; it is believed that lncRNA TP73-AS1 might down-regulate miR-139-3p directly to facilitate Rb cell proliferation [39]. In glioblastoma, TP73-AS1 was up-regulated in tumor tissues and its concentration was associated with a poor prognosis of patients. Furthermore, lncRNA TP73-AS1 facilitates tumor aggressiveness and temozolomide resistance in glioblastoma multiform cancer stem cell (gCSC) [40]. Moreover, Hu et al. [41] analyzed the expression of miR-194 and TP73-AS1 in a great number of healthy tissue samples and cancer cell lines and concluded that the TP73-AS1 and miR-941 duo represent an abnormal case of the exceedingly rapid progression of noncoding modulators managing cell proliferation, migration, and tumorigenesis.

However, there are still some existing problems among the previous studies. First, the function of TP73-AS1 has been well studied in many cancers, but studies on the function of this lncRNA in many common tumors, such as cervical cancer and prostate cancer, are still lacking. Second, the mechanism of the molecular signaling pathways in CCA are not clear. Third, the evaluation of TP73-AS1 in human cancers is very limited. Additionally, the precise mechanisms of this lncRNA contributing to drug resistance remain largely unknown. Noteworthy, studies on the expression of TP73-AS1 in glioma and CRC tissues have shown contradictory results, probably due to diverse functions of different splice variants of TP73-AS1 or sex-related differences between samples. Further researches should be done to find other methods of detection, identify the complex mechanism of regulating responses to chemotherapy, and a larger cohort of cancer samples should be included in the study so that the reasons for the inconsistent research results in the same tumor type might be identified and TP73-AS1 can be used as a biomarker for early diagnosis and therapy of cancer in the clinic.

## Abbreviations

BC: breast cancer
BDH2: butyrate dehydrogenase 2
CCA: cholangiocarcinoma
ccRCC: clear cell renal cell carcinoma
ceRNA: competitive endogenous RNA
CRC: colorectal cancer
EMT: epithelial-to-mesenchymal transition
ESCC: esophageal squamous cell carcinoma
GC: gastric cancer
HCC: hepatocellular carcinoma
HMGB1: high mobility group box 1 protein
LAD: lung adenocarcinoma
lncRNA: long non-coding RNA
miRNA: microRNA
NSCLC: non-small cell lung cancer
OS: overall survival
PDAM: p53-dependent apoptosis modulator
RAGE: receptor for advanced glycation end product
Rb: retinoblastoma
RNA Pol II: RNA polymerase II
SDAD1: SDA1 domain containing 1
TNM: tumor lymph node metastasis
TP53: tumor protein 53
TP73-AS1: P73 antisense RNA 1T
TWIST1: twist family bHLH transcription factor 1
5-FU: 5-Fluorouracil
